# THE ROLE OF SOCIAL ENTERPRISE ORGANIZATIONS IN SUPPORTING FAMILY CARERS OF OLDER PEOPLE LIVING WITH DEMENTIA

**DOI:** 10.1093/geroni/igad104.0342

**Published:** 2023-12-21

**Authors:** Maria Cheshire-Allen, Abigail Davies

**Affiliations:** Swansea University, Swansea, Wales, United Kingdom; Swansea University, Swansea, Wales, United Kingdom

## Abstract

This paper aims to present findings from a primary qualitative study that explored the role of social enterprise organisations (SEOs) in delivering co-produced support for family carers of older people living with dementia (PLWD). Defined in the UK, as businesses with a social or environmental purpose, SEOs are increasingly recognised as occupying a unique space in the landscape of adult social care delivery. In Wales, devolved legislation requires public bodies to collaborate with SEOs - this provision is intended to create a mixed economy of care with co-produced user-led services. To date, however, there is little firm evidence of SEOs delivering co-produced care solutions within the policy framework in Wales, which can be understood to be particularly permissive of SEO involvement, or of how the sector is involved in the delivery of support for family carers of older PLWD. Findings gathered through five focus groups with a purposive sample of family carers, and SEO care providers in South Wales, show how co-produced family carer support programmes are navigated and enacted, as well as how the individual agency of service providers, PLWD, and their carers are involved in these processes. Adopting an ethics of care approach, and utilising reflexive thematic analysis, key themes identified include competition between and within organisations, parochialism, capacity, and organisational barriers. This study highlights implications for policy and practice related to the rhetoric of co-produced support services for family carers, SEOs’ views on delivering that support, and family carers’ experiences of navigating and receiving SEO-delivered services.

